# Characteristics of Microbiota in Different Segments of the Digestive Tract of *Lycodon rufozonatus*

**DOI:** 10.3390/ani13040731

**Published:** 2023-02-17

**Authors:** Yongquan Shang, Huaming Zhong, Gang Liu, Xibao Wang, Xiaoyang Wu, Qinguo Wei, Lupeng Shi, Honghai Zhang

**Affiliations:** 1 College of Life Sciences, Qufu Normal University, Qufu 273165, China; 2 College of Biology and Food, Shangqiu Normal University, Shangqiu 476000, China

**Keywords:** *Lycodon rufozonatus*, microbial diversity, different gut segments, digestive tract, functional analysis, gut microecosystem

## Abstract

**Simple Summary:**

Microorganisms are widely distributed in the skin, respiratory tract, digestive tract, external auditory tract, and urogenital tract of animals, with the highest microbiota abundance in the digestive tract. Considering the vital role of intestinal microbiota in nutrient metabolism, immunity, neuroregulation, and curing of disease, animal intestinal microbiota have become a research hotspot in conservation biology. The intestinal microbiota of mammals are dominated by Firmicutes and Bacteroidetes. The avian gut microbiota are similar to those of mammals at the phylum level and primarily consist of Bacteroidetes, Firmicutes, and Proteobacteria. Proteobacteria and Firmicutes dominated the bacterial communities of the insect gut. However, relatively little research has been conducted on reptiles, particularly snakes. In this study, the composition of the intestinal microbial community and its ecological adaptation were investigated in the different segments of the gastrointestinal tract of *Lycodon rufozonatus* specimens. We found that microbial diversity was higher in the stomach than that in the small and large intestines, and the microbes were involved in metabolic processes. Our results provide insights into the comprehensive understanding of the evolution and ecology of snakes and the formulation of conservation measures for these animals.

**Abstract:**

The gastrointestinal tract of animals contains microbiota, forming a complex microecosystem. Gut microbes and their metabolites can regulate the development of host innate and adaptive immune systems. Animal immune systems maintain intestinal symbiotic microbiota homeostasis. However, relatively few studies have been published on reptiles, particularly snakes, and even fewer studies on different parts of the digestive tracts of these animals. Herein, we used 16S rRNA gene sequencing to investigate the microbial community composition and adaptability in the stomach and small and large intestines of *Lycodon rufozonatus.* Proteobacteria, Bacteroidetes, and Firmicutes were most abundant in the stomach; Fusobacteria in the small intestine; and Proteobacteria, Bacteroidetes, Fusobacteria, and Firmicutes in the large intestine. No dominant genus could be identified in the stomach; however, dominant genera were evident in the small and large intestines. The microbial diversity index was significantly higher in the stomach than in the small and large intestines. Moreover, the influence of the microbial community structure on function was clarified through function prediction. Collectively, the gut microbes in the different segments of the digestive tract revealed the unique features of the *L. rufozonatus* gut microbiome. Our results provide insights into the co-evolutionary relationship between reptile gut microbiota and their hosts.

## 1. Introduction

Interactions between the intestinal microbes and the genomes of vertebrate hosts facilitate adaptation to extreme or new environments [1,2,3]. The gut microbes and their respective animal hosts have co-evolved over a long period to achieve equilibrium and reciprocity [4,5,6]. However, most studies on the digestive tract microbiome have focused on mammals [7,8,9], fish [10], and birds [11,12]. For example, the clustering behavior of the steppe vole (*Lasiopodomys brandtii*) can increase the richness and diversity of its gut microbiota, reduce the abundance of harmful bacteria, and increase the content of beneficial bacteria and metabolites, such as short-chain fatty acids, which provide energy sources for the host. This suggests that the gut microbiota–gut-brain system can regulate the adaptability of animals to cold environments [8]. Zhang et al. (2016) studied the gut microbes of yak (*Bos grunniens*), Tibetan sheep (*Ovis aries*), cattle (*Bos taurus*), and sheep (*Ovis aries*) and found that gut microbes co-evolve to help their host adapt to the extreme plateau environment [13]. Xiao et al. (2021) revealed that zebrafish (*Danio rerio*) adapt to environmental changes by controlling the ecological succession of the gut microbiota during development [14,15]. However, studies on the microbes in the digestive tracts of reptiles are relatively limited. Zhang et al. (2021) found that Bacteroidetes, Firmicutes, and Proteobacteria were the three primary phyla in the gut microbiota [16] and that the diet and lifestyle could affect the gut microbiota in an oviparous lizard (*Calotes versicolor*) of different genders [17]. In addition, Montoya-Ciriaco et al. (2020) found that the diet had an effect on the intestinal microorganisms of the mesquite lizard (*Sceloporus grammicus*) (Wiegmann, 1828) at different altitudes [18].

Among vertebrates, the snake’s ability to endure food scarcity may be the key to its survival [19]. Snakes have extreme fasting cycles; some can fast for up to a year. They can simultaneously regulate energy supply and demand during food shortages, with extreme predation flexibility and a long hunger tolerance time [20]. After ingesting their prey, snakes can immediately recover their ability to digest and absorb food; this requires efficient adaptation on the part of the snake as well as its gut microbes. Studies have revealed that animals with different dietary habits have varied gut microbiota compositions [21]. The microbial diversity of herbivores, omnivores, and carnivores decreases from high to low [22]. In recent years, there has been considerable research on the gut flora of carnivores but relatively little research on snakes. Snakes are strict carnivores; their method for food processing is simple, that is, swallowing food wholly [23]. Therefore, the gastrointestinal microbes of snakes may have a unique community structure and function and co-operate with the host to adapt to their carnivorous feeding methods.

The characteristics of the intestinal microbial structure of animals can provide a theoretical basis for disease diagnosis, which is crucial for the conservation and management of endangered species [24,25,26]. *Lycodon rufozonatus* belongs to the subfamily Colubridae and genus *Dinodon*, which are widely distributed in most parts of China [27]. It is listed as a near-threatened and vulnerable species in the 2013 IUCN Red List of Threatened Species and the Chinese Biodiversity Red List [28,29]. Therefore, studying the composition, diversity, and phylogeny of the intestinal microbiota of this species is necessary to develop comprehensive protection measures. In addition, physiological changes, including the chemistry and nutrient levels, have different effects on bacterial community composition, which means that stool samples do not accurately reflect the entire microbial components of the digestive tract or the microbial structure and function in different segments of the digestive tract [30].

In the present study, we used the high-throughput sequencing to analyze the structure and potential functions of bacterial communities in the small and large intestines of *L. rufozonatus*. These results enrich our understanding of the snake gut microecology and their adaptive co-evolution with the host.

## 2. Materials and Methods

### 2.1. Sample Collection

*L. rufozonatus* was collected from the Beicheng snake and scorpion-breeding farm in Weifang, China, in November 2019. Fifteen healthy adult snakes of the same body length raised under the same environmental conditions were screened. No antibiotics or other drugs that can affect the intestinal microbes were administered to the snakes for 2 months before collection. After 30 days of fasting, a dose of lidocaine hydrochloride was injected into the brain for euthanasia, immediately after which an abdominal incision was made and the digestive tract was removed. The digestive tract contents were collected via sterile swabs, transferred into sterile vials, immediately frozen in liquid nitrogen, and stored at −80 °C until DNA extraction.

### 2.2. DNA Extraction and Polymerase Chain Reaction (PCR) Amplification

DNA was extracted using a QIAamp^®^ Fast DNA Stool Mini Kit (QIAGEN, Hilden, Germany) according to the manufacturer’s protocol. We amplified the V3–V4 region of the bacterial 16S rRNA genes. PCR amplification was performed using the bacterial universal primers 341F and 806R. PCRs were performed in triplicate, with each 30 μL reaction mixture containing 15 µL of Phusion Master Mix (2×), 1.5 μL of each primer (2 μM), and 10 μL of template DNA. The PCR amplification conditions were 98 °C for 3 min, followed by 35 cycles of 98 °C for 45 s, 50 °C for 30 s, and 72 °C for 30 s, and a final extension at 72 °C for 7 min. To ensure a high efficiency and accuracy of amplification, the PCR products were detected using 2% agarose gel electrophoresis. According to the PCR products obtained, the target bands were recovered using a gel recovery kit provided by QIAquick^®^ Gel Extraction Kit (QIAGEN).

### 2.3. Library Construction and High-Throughput Sequencing

A TruSeq DNA PCR-free sample preparation kit (Illumina, San Diego, CA, USA) was used to construct libraries, which were then quantified using Qubit and Q-PCR. Subsequently, Illumina NovaSeq 6000 (PE250; Illumina) was used for on-machine sequencing.

### 2.4. Sequencing Data Processing

Data from each sample were separated from the off-machine data according to the barcode sequence and PCR primer sequences. FLASH software (V1.2.7, http://ccb.jhu.edu/software/FLASH/, accessed on 24 February 2020) was used to splice the reads [31]. The sequences obtained were raw tags [32], which were processed via strict filtering to obtain high-quality tag data (clean tags) as described in QIIME (V1.9.1, http://qiime.org/scripts/split_libraries_fastq.html, accessed on 24 February 2020) [33]. The clean tag sequences were compared with the VSEARCH (https://github.com/torognes/vsearch/, accessed on 24 February 2020) database to detect chimeric sequences [34]. Finally, chimeric sequences were removed to obtain the final effective tags.

### 2.5. Operational Taxonomic Unit Clustering and Species Annotation

Operational taxonomic units (OTUs) with ≥97% similarity were used to cluster the effective tags using the UPARSE (V7.0.1001) pipeline [35]. Mothur software was used for comparison with the SSU rRNA database of SILVA (http://www.arb-silva.de/, accessed on 25 February 2020) to obtain species annotation information [36,37]. Multiple sequence alignments of the OTUs were performed using MUSCLE (http://www.drive5.com/muscle/, accessed on 25 February 2020) software to generate evolutionary trees [38].

### 2.6. Diversity Analysis

Species diversity and complexity were calculated using QIIME software (V1.9.1) [33]. Rarefaction curves were drawn using R software (V2.15.3) (R Foundation for Statistical Computing, Vienna, Austria). For the alpha diversity analysis, the Wilcoxon rank-sum and Tukey’s tests were used to compare the significance of the differences between groups. The UPGMA sample clustering tree was constructed using the QIIME software (V1.9.1), and the nonmetric multidimensional scaling (NMDS) of the Bray–Curtis distances was generated using the vegan package (V2.15.3) of R software. The analysis of molecular variance tests was conducted using Mothur software. R software was used to perform *t*-tests between groups and plot the species with significant differences.

### 2.7. Indicator Species Analysis

Linear discriminant analysis (LDA) (effect size score > 4) and MetaStats analysis were used to explore the microbiota with significant differences among the groups [39]. Metastatic analysis was performed using R software at the phylum and genus levels. The *p*-values were obtained using a permutation test between groups and then modified using the Benjamini and Hochberg false discovery rate method to obtain the q-values [40].

### 2.8. Function Prediction

Trees based on the OTUs were generated using the Greengene database; Phylogenetic Investigation of Communities by Reconstruction of Unobserved States (PICRUSt) was used to map the sequencing composition into a database to predict the metabolic function of the microbiota.

## 3. Results

### 3.1. Sequencing of the Microbiota in the Gastrointestinal Tract of L. rufozonatus

In the study, we collected 15 *L. rufozonatus* samples from three gut segments (stomach, small intestine, and large intestine) under fasting conditions. Owing to the low gastrointestinal content in snakes during sampling, 14 samples (four from the stomach, three from the small intestine, and seven from the large intestine) were successfully collected and sequenced using the Illumina NovaSeq 6000 sequencing platform. After data quality control, 1,062,719 clean tags were obtained (278,089 for the stomach, 218,229 for the small intestine, and 566,331 for the large intestine). After chimera filtering, 873,406 effective tags were obtained for subsequent analysis (including 244,838, 186,950, and 441,618 tags for the stomach, small intestine, and large intestine, respectively). The average sequence length was 409.4 bp, which covered the full length of the V3–V4 region. The effective sequence of all samples was 67.82 ± 4.75%, which met the criteria for the follow-up statistical analysis (Table 1). As shown by the rarefaction curve (Figure S1), when the amount of sequencing data reaches 30,000 reads, the sample curve gradually flattens and finally becomes stable; this indicates that the amount of sequencing reflects the diversity of the microbiome, thereby ensuring the reliability of the subsequent analysis.

### 3.2. Bacterial Diversity in the Gastrointestinal Tract

**Figure 1:**
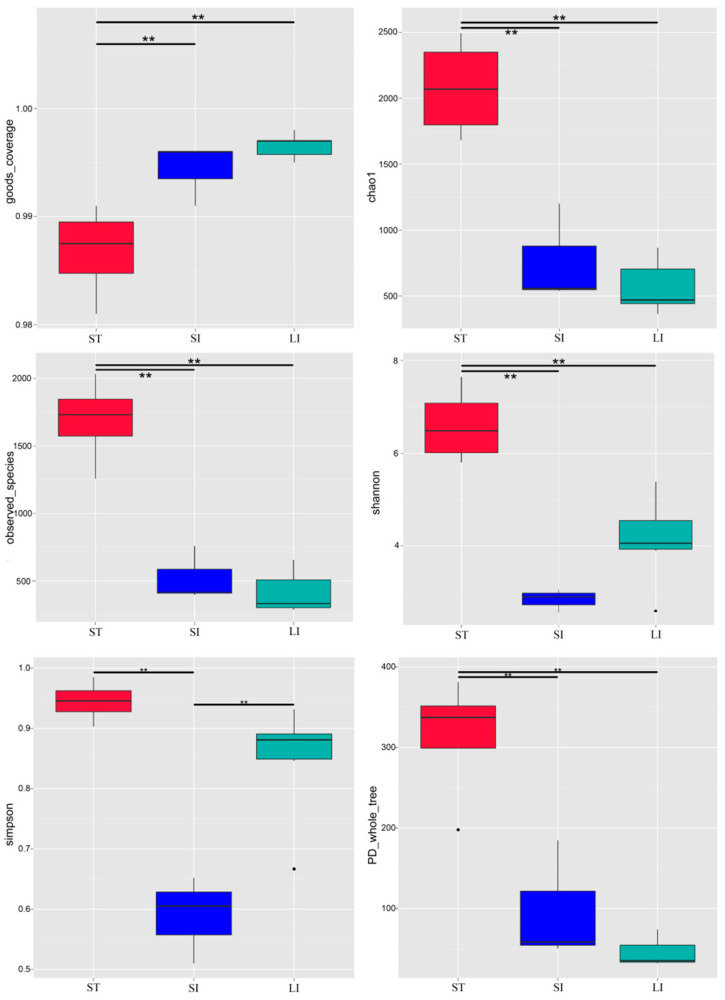
Alpha diversity analysis of the different segments of the gastrointestinal tract of *L. rufozonatus*. Abbreviations: LI, large intestine; SI, small intestine; ST, stomach; **: *p* < 0.01.

The alpha diversity indices of the different samples were calculated (Table S1, Figure 1). Among them, the goods-coverage results were 99%, indicating that sufficient 16S rRNA gene sequences were extracted from the intestine of *L. rufozonatus* to optimally evaluate bacterial diversity. The average number of observed species in the stomach (1689.00 ± 323.21) was significantly higher (*p* < 0.01) than that in the small (525.00 ± 202.01) and large intestines (429.14 ± 149.56). However, no significant difference was observed in species number between the small and large intestines (*p* > 0.05). The Shannon index of the stomach was significantly higher (*p* < 0.01) than that of the small and large intestines; similarly, no significant difference was found between the large and small intestines (*p* > 0.05). The Simpson’s index of the stomach and large intestine was significantly higher (*p* < 0.01) than that of the small intestine, indicating the highest microbial diversity in the stomach and the lowest in the small intestine. The average level of the phylogenetic diversity whole-tree index in the stomach was significantly higher (*p* < 0.01) than that in the small and large intestines.

**Figure 2:**
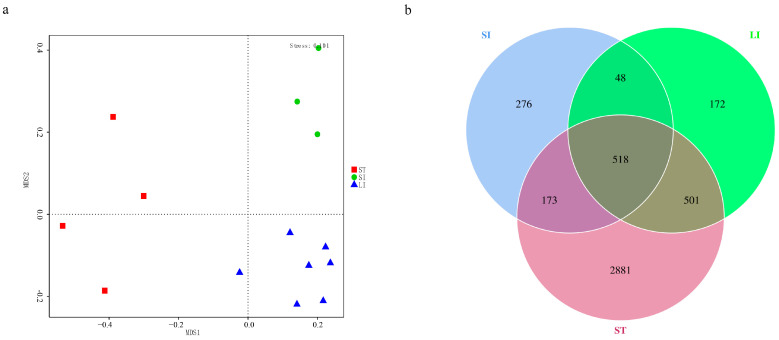
(**a**) Non−metric multidimensional analysis of the microbial community structures of the gut microbiota in the three segments of the *L. rufozonatus* digestive tract. (**b**) Venn diagram of gut microbiota in *Lycodon rufozonatus* at the operational taxonomic unit (OTU). Abbreviations: LI, large intestine; SI, small intestine; ST, stomach.

The β diversity of gut microbial composition was evaluated by NMDS and Venn diagram of the gut microbiota (Figure 2). NMDS analysis revealed significant differences in the microbial composition among the different segments based on the Bray–Curtis distance (Figure 2a). To explore the commonality and particularity of the microbes, Venn diagrams were used to calculate the number of OTUs shared by the different segments. We found that the number of core OTUs shared by the three segments was 518, whereas the number of unique OTUs in the stomach group was the largest (2881) (Figure 2b).

### 3.3. Bacterial Composition and Structure of the Gastrointestinal Tract

**Figure 3:**
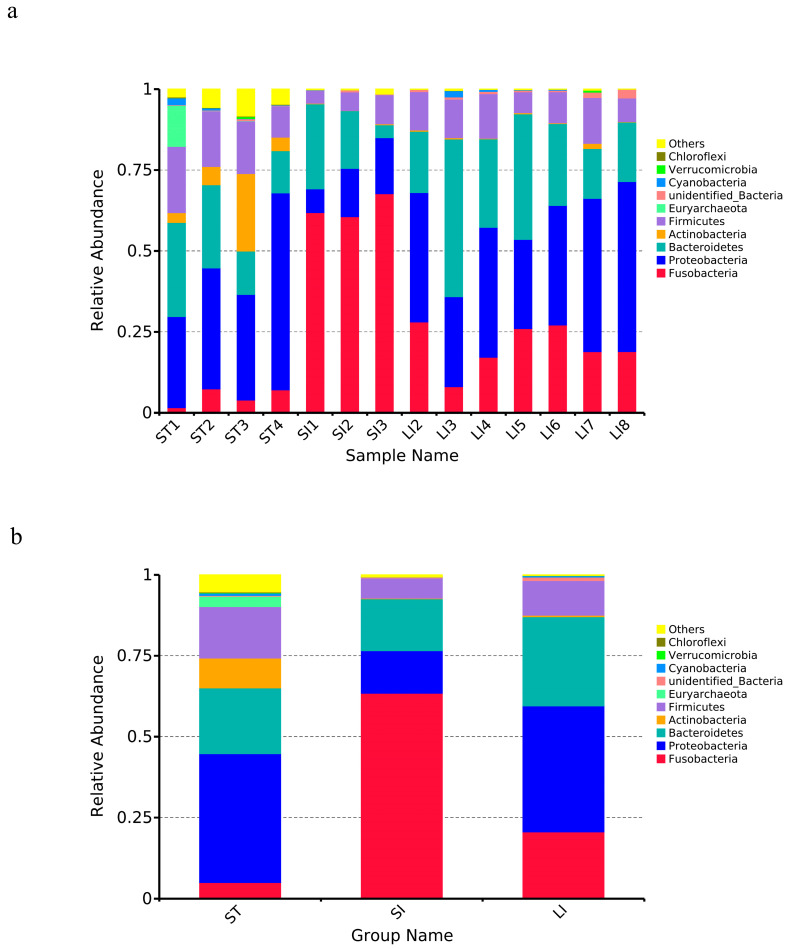
Microbial composition among the different samples (**a**) and segments (**b**) at the phylum level. The top 10 abundant taxa are shown. Abbreviations: LI, large intestine; SI, small intestine; ST, stomach.

To study the species composition of each sample, the effective tags of all samples were clustered by OTU with 97% similarity. The OTU sequences were annotated with 36 phyla, 55 classes, 118 orders, 223 families, 517 genera, and 390 species. At the phylum level, all segments had high abundances of Proteobacteria, Firmicutes, Fusobacteria, Bacteroidetes, and Actinobacteria [41] (Figure 3a). Among the different segments, the stomach had a relatively high abundance of Proteobacteria (39.75 ± 14.56%), Bacteroidetes (20.32 ± 8.32%), Firmicutes (15.92 ± 4.53%), Actinomycetes (9.18 ± 9.90%), and Fusobacteria (3.21 ± 2.76%). The small intestine had a high abundance of Fusobacteria (63.41 ± 3.77%), Bacteroidetes (16.04 ± 11.27%), Proteobacteria (13.18 ± 5.23%), Firmicutes (6.25 ± 2.57%), and Actinomycetes (0.18 ± 0.12%%). Finally, the large intestine had a high abundance of Proteobacteria (38.90 ± 9.28%), Bacteroidetes (27.56 ± 12.15%), Fusobacteria (20.63 ± 7.11%), Firmicutes (10.72 ± 3.05%), and Actinomycetes (1.05 ± 0.50%). The bacterial abundance in the different sections of the gastrointestinal tract varied greatly, showing spatial heterogeneity (Figure 3b). As shown in Figure 3, the dominant microbiota of the stomach and large intestine were similar, primarily from the phyla Proteobacteria, Bacteroidetes, and Firmicutes. The stomach was specifically enriched with Actinomycetes compared to the other two segments; however, the small intestine contained more *Fusobacterium* species than the other segments.

**Figure 4:**
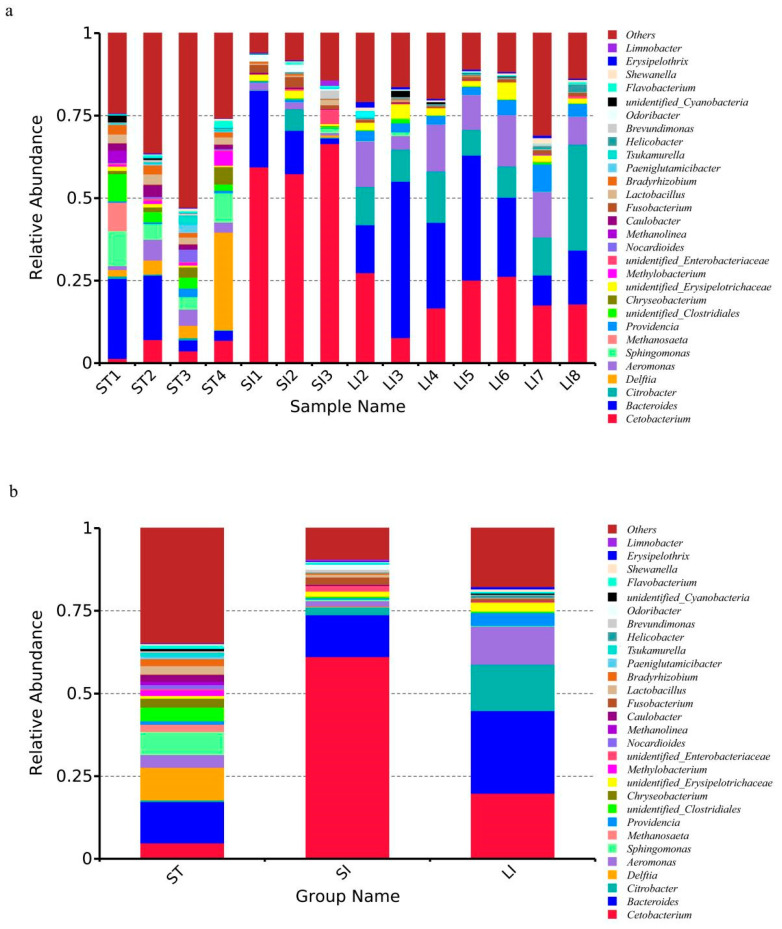
Microbial composition of different samples (**a**) and segments (**b**) at the genus level. The top 30 most abundant taxa are shown. Abbreviations: LI, large intestine; SI, small intestine; ST, stomach.

At the genus level, 517 genera were identified in all three segments (Figure 4). The top 10 bacterial genera with the highest abundance in the stomach were as follows: other (34.74 ± 13.18%) *Bacteroides* (12.50 ± 11.00%), *Delftia* (9.85 ± 13.16%), *Sphingomonas* (7.03 ± 3.26%), *Cetobacterium* (4.81 ± 2.74%), unidentified Clostridiales (4.14 ± 2.82%), *Aeromonas* (3.88 ± 2.22%), *Chryseobacterium* (2.69 ± 1.96%), *Lactobacillus* (2.45 ± 0.48%), and *Methanosaeta* (2.16 ± 4.29%). The top 10 most abundant genera in the small intestine were *Cetobacterium* (61.15 ± 4.79%), *Bacteroides* (12.65 ± 10.75), other (9.38 ± 4.30%), *Citrobacter* (2.32 ± 3.61%), *Fusobacterium* (2.26 ± 1.04%), *Aeromonas* (1.72 ± 0.84), *Odoribacter* (1.62 ± 0.95%), unidentified Enterobacteriaceae (1.59 ± 2.30%), unidentified Erysipelotrichaceae (1.58 ± 0.89%), and *Brevundimonas* (0.85 ± 1.37%). The top 10 most abundant genera in the large intestine were *Bacteroides* (25.01 ± 13.58%), *Cetobacterium* (19.85 ± 6.99%), other (17.71 ± 6.95%), *Citrobacter* (13.94 ± 8.35%), *Aeromonas* (11.54 ± 4.08%), *Providencia* (3.89 ± 2.06%), unidentified Erysipelotrichaceae (2.71 ± 1.46%), *Helicobacter* (0.78 ± 0.76%), *Shewanella* (0.77 ± 0.51%), and *Fusobacterium* (0.77 ± 0.31%) (Table 2). The above data show, according to abundance, that the dominant genera in the stomach were not apparent; however, those in the intestines were more evident. Specifically, *Cetobacterium* and *Bacteroides* were dominant in the small intestine, whereas *Bacteroides*, *Cetobacterium*, *Citrobacter*, and *Aeromonas* were abundant in the large intestine.

### 3.4. Differences in the Gastrointestinal Microbiome across Segments

**Figure 5:**
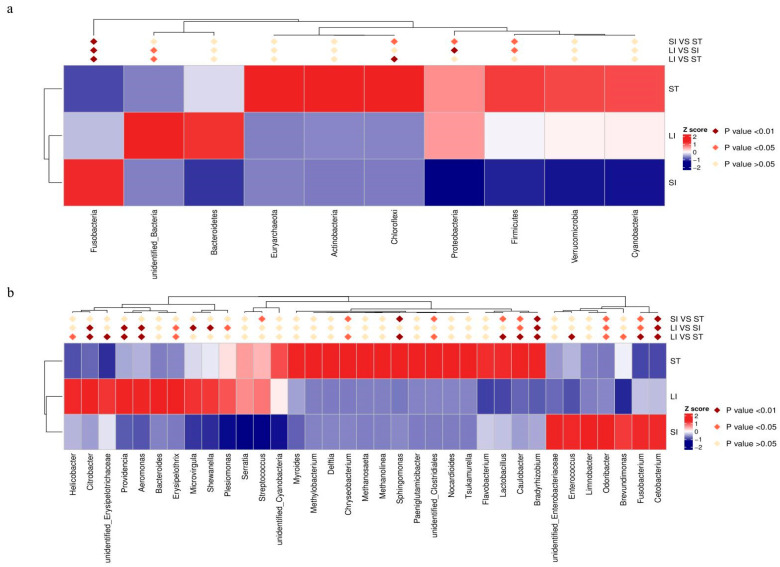
Statistical heat map of the significantly different species at the phylum and genus levels. (**a**) Top 10 species with significant differences at the phylum level. (**b**) Top 35 species with significant differences at the genus level. Abbreviations: LI, large intestine; SI, small intestine; ST, stomach.

The MetaStat method was used to draw a heat map of species abundance data between groups at the phylum and genus levels to show the abundance and significance of different species in the different segments of the gastrointestinal tract. The abundances of Choroflexi, Proteobacteria, and Fusobacteria in the large intestine were significantly higher than those in the small intestine (*p* < 0.05); the abundance of Choroflexi in the stomach was significantly higher than that in the large intestine (*p* < 0.01); the abundance of Fusobacteria in the small intestine was significantly higher than in the stomach and large intestine (*p* < 0.01); and the abundance of Bacteroidetes in the large intestine was significantly higher than that in the stomach and small intestine (*p* < 0.05) (Figure 5a). At the genus level, *Chryseobacterium*, *Sphingomonas*, unidentified Clostridiales, *Lactobacillus*, and *Caulobacter* were significantly more abundant in the small and large intestines (*p* < 0.05) than in the stomach in this instance. *Odoribacter*, *Fusobacterium*, and *Cetobacterium* were significantly more abundant in the small intestine than in the stomach and large intestines (*p* < 0.05). The abundance of *Citrobacter*, *Providencia*, *Aeromonas*, and *Erysipelothrix* in the large intestine was significantly higher than that in the stomach and small intestine (*p* < 0.05). The abundance of *Helicobacter* and Erysipelotrichaceae in the large intestine was significantly higher than that in the stomach, whereas that of *Microvirgula*, *Shewanella*, and *Plesiomonas* in the large intestine was significantly higher than that in the small intestine (Figure 5b).

**Figure 6:**
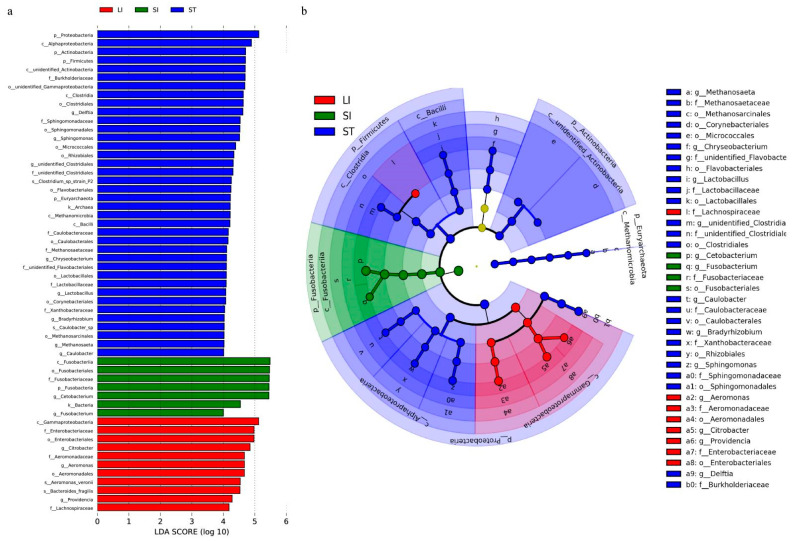
Biomarker analysis of microbial communities from the different segments of the gastrointestinal tract. (**a**) Differentially abundant taxa. The length of the histogram represents the impact of the different species (LDA > 4). (**b**) Cladogram showing the phylogenetic structures of the microbiota. Abbreviations: LI, large intestine; SI, small intestine; ST, stomach.

LDA was used to compare the communities that could be considered different biomarkers. Specifically, 38 biomarkers were identified for the stomach, 7 for the small intestine, and 11 for the large intestine (Figure 6). The most differentially abundant bacteria in the stomach were *Lactobacillus* and *Clostridia* in Firmicutes, *Sphingomonas* and *Delftia* in Proteobacteria, *Corynebacterium* and Micrococcaceae in Actinomycetes, and *Methanosaeta* in Euryarchaeota. In contrast, those in the small intestine were *Cetobacterium* and *Fusobacterium spp*. The species biomarkers in the large intestine primarily comprise the genera *Aeromonas*, *Citrobacter*, *Providencia*, and those belonging to the family Lachnospiraceae.

### 3.5. Microbial Function Prediction

**Figure 7:**
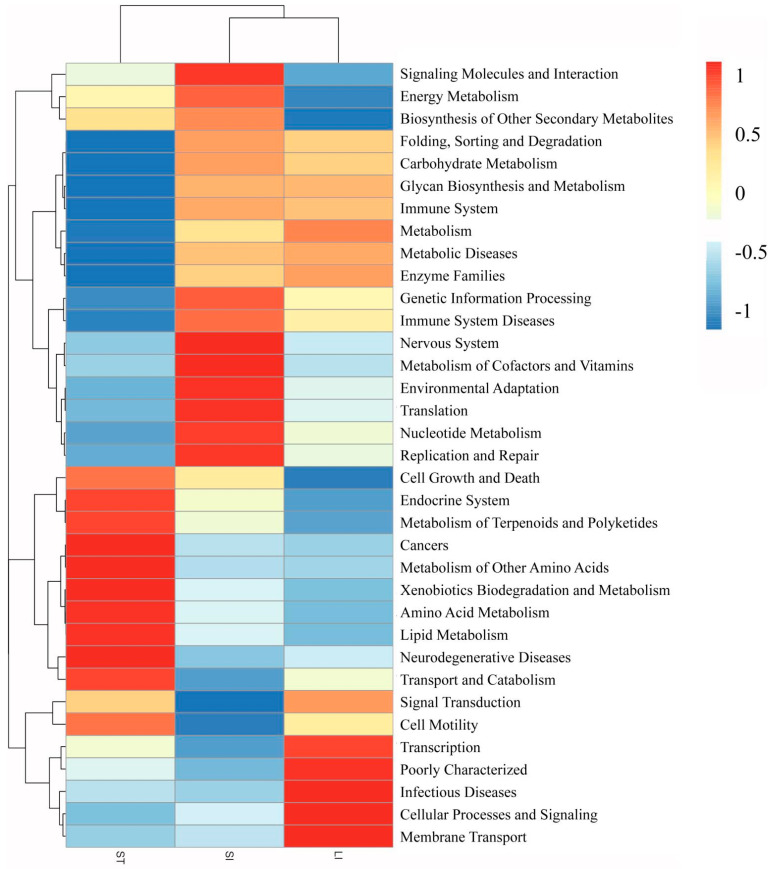
Heatmap of the hierarchy cluster results for the functional diversity of microbiota in the different segments of the gastrointestinal tract. Abbreviations: LI, large intestine; SI, small intestine; ST, stomach.

To study the possible functional pathways of bacterial microbiota in various segments of the gastrointestinal tract, functional prediction was carried out using the PICRUSt software (version (1.0.0), created by Lingille et al., in 2013, USA) based on the KEGG database. A heat map of functional clustering at level two is shown in Figure 7. The most abundant functions in the stomach include the metabolism of terpenoids and polyketides, cancers, metabolism of other amino acids, xenobiotic biodegradation and metabolism, amino acid metabolism, lipid metabolism, neurodegenerative diseases, transport, and catabolism. The most common functions in the small intestine include signaling molecules and interactions, the nervous system, metabolism of cofactors and vitamins, environmental adaptation, translation, nucleotide metabolism, replication, and repair. The most common functions in the large intestine involve transcription, infectious diseases, cellular processes and signaling, and membrane transport. The small and large intestines were observed to cluster together, as their functional pathways have partially similar color modules. The corresponding functions of these modules, such as glycan biosynthesis and metabolism, immunity, metabolism, and enzyme families, had similar positions in the two segments of the intestine. The functional pathways of the microbiota in the stomach were remarkably different from those in the small and large intestines.

**Figure 8:**
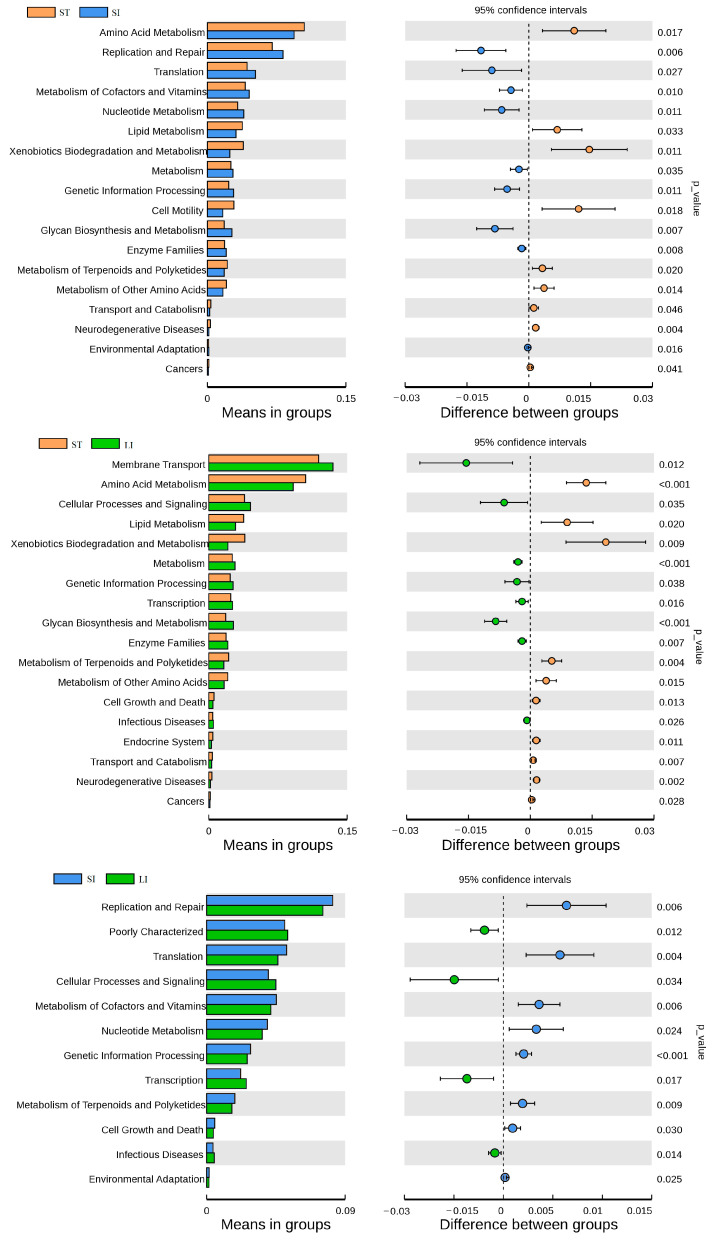
Comparison of the microbial functions (level two, top 20) that were significantly different among the three segments of the gastrointestinal tract. Abbreviations: LI, large intestine; SI, small intestine; ST, stomach.

The significance of the functional differences at level two of the gastrointestinal tract was detected using t-tests based on the database annotation results (Figure 8). The results showed, at level two, 18 pathways significantly differed among the stomach, small intestine, and large intestine, among which 8 and 5 pathways were involved in metabolism, respectively. In total, 3 of the 12 functional pathways with significant differences between the small and large intestines were related to metabolism.

## 4. Discussion

Vertebrates have a close and complex relationship with the microbial communities in their gastrointestinal tract [42,43,44,45]. The typical composition and structure of the microbiota can reflect the function of the gastrointestinal tract and can be used to evaluate the health state or diagnose [46]. Previous studies have revealed that the gut microbiota of wild mice promote host fitness and improve disease resistance [47]. As in other vertebrates, microbes in different parts of the digestive tract play an important role in the growth of snakes. Therefore, studying snake intestinal microbes has become an important element in snake conservation research. In this study, we conducted the first comprehensive analysis of the gastrointestinal microbiota of *L. rufozonatus* using high-throughput sequencing technology and revealed changes in the community structure and function of the intestinal microbiota in the different segments of its digestive tract.

The dominant gut microbiota in snakes vary greatly across species. Previous studies have revealed that the dominant phyla in the gut of *Crotalus horridus* are Proteobacteria and Firmicutes; however, the abundance of Proteobacteria in *Crotalus scredus* is as high as 85.0% [48]. Bacteroidetes, Firmicutes, and Proteobacteria are the dominant bacterial phyla in the intestinal tract of *Agkistrodon piscivorus* [49]. The five most abundant phyla in *Deinagkistrodon acutus* are Bacteroidetes, Proteobacteria, Firmicutes, Fusobacteria, and Actinobacteria [50]. *Naja atra*, *Ptyas mucosus*, and *Elaphe carinata have the same dominant* microbiota *as Deinagkistrodon acutus*, which were Bacteroidetes, Proteobacteria, Firmicutes, Fusobacteria, and Actinobacteria [50]. Similar microbiota structures have been found in the oviparous lizard (*Calotes versicolor*) and marine iguana (*Amblyrhynchus cristatus*), with Firmicutes, Bacteroidetes, and Proteobacteria as the dominant phyla in their gut microbiota [16,51]. In this study, we found that the phyla with high abundance in all the segments of the gastrointestinal tract of *L. rufozonatus* were Proteobacteria, Firmicutes, Fusobacteria, Bacteroidetes, and Actinobacteria. Specifically, Proteobacteria, Bacteroidetes, and Firmicutes were predominant in the stomach; Fusobacteria was the most abundant in the small intestine; and Proteobacteria, Bacteroidetes, Fusobacteria, and Firmicutes were dominant in the large intestine. Although there are differences in the core phyla found in the different segments, the results of studies on the dominant intestinal microbiota of *Crotalus horridus*, *Agkistrodon piscivorus*, *Naja atra, Ptyas mucosus*, *Calotes versicolor*, and *Amblyrhynchus cristatus* are similar [16,48,49,50,51]. However, Bacteroidetes and Firmicutes are the dominant phyla in the intestinal tract of mammals; however, the dominant phyla in non-passerine birds are Firmicutes, Proteobacteria, Actinobacteria, and Bacteroidetes, reflecting the influence of phylogenetic relationships on the intestinal microorganisms in animals [21,52].

The different segments of the gastrointestinal tract of snakes are highly specialized regions in which the symbiotic bacteria of the stomach and small intestine play an important role in digestion and absorption, respectively. Significant differences were observed in the distribution and structure of the bacterial communities between the stomach and small and large intestines. The alpha diversity index showed that the bacterial diversity and abundance in the stomach were significantly higher than those in the large and small intestines. Snakes are carnivores that devour whole prey, including fur, feathers, bones, and even undigested food from their prey’s gut [53]. As digestion in snakes is relatively slow, the food remains in their stomach for a long time. The abundance of bacteria in the stomach of *L. rufozonatus* was higher than that in the intestinal segments, consistent with a study on herbivorous Bactrian camels [54]. Camels retain food particles in the rumen considerably longer than other large herbivores. Therefore, we speculated that the alpha diversity of the digestive tract microbes is related to the rate of digestion. 

Previous studies have revealed that Proteobacteria and Firmicutes are dominant in the stomach, small intestine, and colon of *C. horridus*, and their primary metabolic pathway is carbohydrate metabolism [48]. In *A. piscivorus*, Bacteroidetes is the dominant phylum in the large intestine, whereas Proteobacteria is dominant in the small intestine and cloaca [49]. In contrast, the gut microbiota of Burmese pythons primarily consist of the members of Bacteroidetes and Firmicutes [55]. In addition, *Bacteroides* are predominant in the large intestine of Burmese pythons during fasting; however, Firmicutes show an overall increase in richness and diversity during digestion [55]. In this study, we found significant differences in the structure of the microbiota in different segments of the gastrointestinal tract, which we speculate was caused by predation on different species. Burmese pythons feed on large prey, such as small mammals and rodents, whereas *L. rufozonatus* feed on frogs, lizards, and fish. Therefore, the type of food plays an important role in intestinal microbe composition.

Proteobacteria is the dominant phylum in the stomach and large intestine but is also present in the small intestine. Proteobacteria accounts for a large proportion of the intestinal microbiota in reptiles. The abundance of Proteobacteria in the guts of *Amblyrhynchus cristatus*, *Liolaemus parvus*, *Liolaemus ruibali*, and *Phymaturus williamsi* ranges from 19.1% to 56.4% [51,56]. The abundance of Proteobacteria in the gut of Burmese pythons is 10.1% [55]. The predominance of Proteobacteria in the gut has also been observed in red-crowned cranes (*Grus japonensis)* [57]. Based on this, some studies have proposed that the gut microbiota of snakes are more similar to those of birds than mammals, which indicates that phylogenetic relationships may play a role in shaping the gut microbiota [49]. 

The composition of intestinal microbiota plays an important role in the digestion and absorption of *L. rufozonatus*. Proteobacteria are facultative anaerobic bacteria that typically degrade and ferment complex sugars. Among these, *Escherichia* may also help the host obtain vitamins [58]. In a healthy gut, Proteobacteria fight infection or inflammation and play a protective role in the immune response [59]. However, Proteobacteria enrichment in the human gut is an indicator of gut microbiota imbalance and is associated with host disease [59]. In addition, the enrichment of Proteobacteria in the gut may indicate an unbalanced and unstable microbial community structure or a diseased state [59]. *Aeromonas*, a genus of Proteobacteria, is found in the stomach and small and large intestines. The genus *Aeromonas* comprises strict aerobes or facultatively anaerobic bacteria that produce acids by breaking down carbohydrates [60]. This genus is typically associated with aquatic environments and is spread via food, people, and animals that come into contact with water [60]. We speculate that the widespread colonization of *Aeromonas* in the gastrointestinal tract of *L. rufozonatus* is consistent with its ecological niche, which is aquatic, as it feeds on frogs, lizards, and fish [61]. 

In addition, Citrobacter, another genus in Proteobacteria, accounts for a proportion of bacteria in the small (2.32%) and large intestines (13.94%). This group of bacteria comprises facultative anaerobes, which are pathogenic to animals and can cause enteritis [62]. *Citrobacter* belongs to the Enterobacteriaceae family, and its enrichment is related to the carnivorous diet of the host [63]. Studies have found that common kestrels (*Falco tinnunculus*), which are strict carnivores, have a high abundance of Enterobacteriaceae in their gut [64]. The food structure of carnivores is high in fat and protein; therefore, as an obligate carnivore, *L. rufozonatus* also has a microbial lineage unique to carnivores.

Bacteroidetes was the second most abundant phylum (16.04–27.56%) in each segment in the gastrointestinal tract of *L. rufozonatus*. *Bacteroides* is the most abundant bacterial genus in the stomach and large intestine and the second most abundant in the small intestine. *Bacteroides* is the most common species of microbial communities in the intestines of timber rattlesnakes (*Crotalus horridus*), cottonmouths (*Agkistrodon piscivorus*), and American freshwater alligators (*Alligator mississippiensis*) [48,49,65]. The genus *Bacteroides* comprises strict anaerobes that can decompose polysaccharides and improve nutrient utilization, thereby promoting digestion and increasing the utilization of complex carbohydrates [66]. It may also contribute to the development of the host intestinal mucosa and immune system and maintain the balance of the intestinal microecology [58,67]. Therefore, *Bacteroides* species is essential for both carnivorous and herbivorous diets.

Fusobacteria is highly abundant in the small intestine of *L. rufozonatus*, similar to that in the American freshwater alligators (*Alligator mississippiensis*) [65]. In contrast, those abundant in other vertebrates are Bacteroidetes, Firmicutes, and Proteobacteria [21,55,68]. Fusobacteria comprises predominantly anaerobic Gram-negative bacilli that metabolize carbohydrates to produce butyrate and provide energy to the intestinal cells [69,70]. Fusobacteria is abundant in humans, and it plays a key role in biofilm development [71]. In addition, Fusobacteria is also involved in the metabolism of amino acids, which may affect the host’s ability to degrade proteins, as reported in the studies of the gut microbiota of alligators (*Alligator mississippiensis*) [65], black vultures (*Coragyps atratus*), turkey vulture (*Cathartes aura*) [72], catfish (*Ictalurus punctatus*), largemouth bass (*Micropterus salmoides*), and bluegills (*Lepomis macrochirus*) [73]. Studies on microbes in the digestive tract of alligators suggest that Fusobacteria may function during the development of digestive organs and the acquisition of nutrients, whereas, in mammals, Firmicutes and Bacteroidetes perform similar functions [65]. Among the Fusobacteria, *Cetobacterium* (61.15%) has the highest abundance in the small intestine and the second highest abundance in the large intestine (19.85%). This is similar to the dominant genera in the microbiomes of carnivorous fish [74], as studies have revealed that *Cetobacterium* and *Halomonas* are the main microflora of carnivorous fish [74,75]. These results further indicate that the structure of the intestinal microbiota of *L. rufozonatus* may be closely related to its diet. We speculated that the accumulation of microbes in the snake’s stomach is mainly related to the position of the snakes in the natural food chain. *L. rufozonatus* preys on fish, leading to the accumulation of specific microbes in the gut of fish.

By comparing the microbial functions of the different segments of the digestive tract, we found that the microorganisms in the stomach are involved in more metabolic processes than those in the intestine. Specifically, the significantly enriched pathways are almost all involved in metabolism, typically those related to amino acids, lipids, terpenoids, and polyketides, and xenobiotic biodegradation. This suggests that the microbiota are important in the construction of the metabolic capacity of the host and are most pronounced in the stomach. These results are also similar to previous studies showing that different intestinal morphologies and physiological and biochemical environments are more likely to influence metabolic function than the diet [22,76]. Although the PICRUSt algorithm has high accuracy, it does not represent absolute accuracy in predicting gene function. Therefore, it will be necessary to use a metagenomic approach in subsequent studies to explore the metabolic effects of the microbiota in the different segments of the digestive tract of *L. rufozonatus*.

## 5. Conclusions

To understand the relationship between digestive tract microbiota and the nutrition and health of *L. rufozonatus*, we aimed to provide a reference for conserving this species. In this study, we used high-throughput sequencing technology and 16S rRNA sequencing to reveal the structure and distribution of bacterial microbiota in different segments of the gastrointestinal tract of *L. rufozonatus* for the first time. The results showed that the microbiota structure was spatially heterogeneous across the different segments of the gastrointestinal tract. At the phylum level, Proteobacteria, Bacteroidetes, and Firmicutes were predominant in the stomach; Fusobacteria was predominant in the small intestine; and Bacteroidetes and Firmicutes were predominant in the large intestine. The dynamic distribution of Proteobacteria and Fusobacteria was evident in the different segments of the digestive tract of *L. rufozonatus*. In addition, the prevalence of *Aeromonas* in the gastrointestinal tract may be related to the fact that *L. rufozonatus* lives in or near aquatic environments and preys on fish. Functional prediction indicated that the microbiota are important in the metabolic capacity of the host. Overall, our findings provide a basis for understanding the complex co-evolutionary relationship between gut microbes and their hosts and will provide an important reference for a comprehensive understanding of the evolution and ecology of snakes and in formulating measures to conserve them.

## Figures and Tables

**Table 1:** Statistics of the sequencing data preprocessing and quality control.

Sample	Raw Tags	Clean Tags	Effective Tags	Average Length (nt)	Effective %	OTU Number	
ST1	77,930	74,107	65,029	406	63.20	1404	1785.50 ± 313.97
ST2	70,079	66,079	56,912	402	64.98	1780	
ST3	74,059	73,020	62,358	378	63.80	2173	
ST4	65,960	64,883	60,539	376	62.48	1785	
SI1	71,633	69,123	58,873	412	72.97	505	617.33 ± 225.56
SI2	73,758	70,630	59,189	413	69.12	470	
SI3	83,937	78,546	68,888	406	60.69	877	
LI1	82,568	79,963	63,326	419	68.42	406	501.71 ± 173.88
LI2	87,412	84,511	65,075	421	67.78	687	
LI3	79,281	77,137	62,150	420	74.34	360	
LI4	78,855	76,421	61,759	417	73.93	588	
LI5	92,005	89,603	61,180	420	63.34	348	
LI6	86,308	83,892	68,675	420	71.38	761	
LI7	76,944	74,804	59,453	422	73.03	362	

Abbreviations: LI, large intestine; SI, small intestine; ST, stomach; OTU, operational taxonomic unit; nt, nucleotide.

**Table 2:** The top 10 bacterial genera in different segments of the digestive tract of *Lycodon rufozonatus*.

Stomach (ST)	Small Intestine (SI)	Large Intestine (LI)
Other (34.74%)	*Cetobacterium* (61.15%)	*Bacteroides* (25.01%)
*Bacteroides* (12.50%)	*Bacteroides* (12.65%)	*Cetobacterium* (19.85%)
*Delftia* (9.85%)	Other (9.38%)	Other (17.71%)
*Sphingomonas* (7.03%)	*Citrobacter* (2.32%)	*Citrobacter* (13.94%)
*Cetobacterium* (4.81%)	*Fusobacterium* (2.26%)	*Aeromonas* (11.54%)
unidentified Clostridiales (4.14%)	*Aeromonas* (1.72%)	*Providencia* (3.89%)
*Aeromonas* (3.88%)	*Odoribacter* (1.62%)	unidentified Erysipelotrichaceae (2.71%)
*Chryseobacterium* (2.69%)	unidentified Enterobacteriaceae (1.59%)	*Helicobacter* (0.78%)
*Lactobacillus* (2.45%)	unidentified Erysipelotrichaceae (1.58%)	*Shewanella* (0.77%)
*Methanosaeta* (2.16%)	*Brevundimonas* (0.85%)	*Fusobacterium* (0.77%)

## Data Availability

The datasets generated for this study can be found in the SRA database of the NCBI database (Accession Number: PRJNA892747).

## Supplementary Materials

The following supporting information can be downloaded at: https://www.mdpi.com/article/10.3390/ani13040731/s1. Figure S1: Rarefaction analysis for the assessment of OTU coverage. Rarefaction curves for different samples (a) and segments (b). Table S1: Statistical analyses of the alpha diversity.
