# Supplementary material for: Characteristics of Microbiota in Different Segments of the Digestive Tract of Lycodon rufozonatus
Source: Animals (Basel). 2023 Feb 17;13(4):731. doi: 10.3390/ani13040731 (PMC9952230; doi:10.3390/ani13040731)
Supplement: Supplementary file 1 [file animals-13-00731-s001.zip › animals-2141704-supplementary.pdf]

Supplementary Material

1 Supplementary Table

Table S1. Statistical analyses of alpha diversity.

	observed_species	shannon	simpson	chao1	goods_coverage	PD_whole_tree
ST	1,689.00±323.21	6.60±0.83	0.95±0.03	2,078.46±382.34	0.989±0.0043	313.51±79.84
SI	525.00±202.01	2.85±0.25	0.59±0.07	765.23±377.02	0.994±0.003	97.83±75.31
LI	429.14±149.56	4.36±0.54	0.88±0.03	585.82±193.74	0.996±0.001	45.96±16.54

2 Supplementary Figure

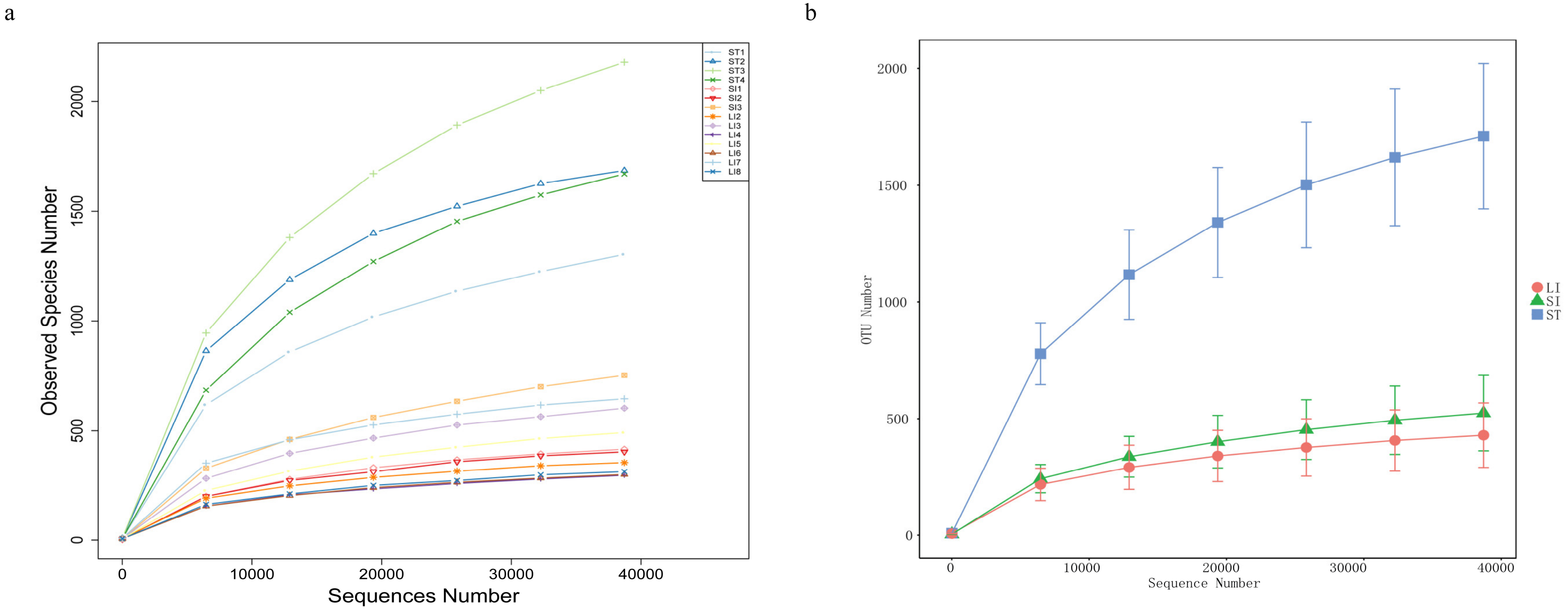

Figure S1. Rarefaction analysis for the assessment of OTU coverage. Rarefaction curves for different samples (**a**) and segments (**b**).
